# Risk factors for acquisition of scrub typhus in children admitted to a tertiary centre and its surrounding districts in South India: a case control study

**DOI:** 10.1186/s12879-019-4299-2

**Published:** 2019-07-26

**Authors:** Winsley Rose, Gagandeep Kang, Valsan Philip Verghese, Sadanandane Candassamy, Prasanna Samuel, John Jude Antony Prakash, Jayaprakash Muliyil

**Affiliations:** 1 Department of Pediatrics, Christian Medical College, Vellore, Tamil Nadu India; 2 Department of Gastrointestinal Sciences, Christian Medical College, Vellore, Tamil Nadu India; 3 Department of Health Research, Vector Control Research Centre, Pondicherry, India; 4 Department of Biostatistics, Christian Medical College, Vellore, Tamil Nadu India; 5 Department of Microbiology, Christian Medical College, Vellore, Tamil Nadu India; 6 Present Address: Translational Health Science and Technology Institute, Faridabad, Haryana India

**Keywords:** Scrub typhus, Children, Risk factors, Mites

## Abstract

**Background:**

Scrub typhus is a mite borne zoonosis common in the tropics with no good preventive strategy. Children are also affected leading to considerable morbidity and mortality. We conducted a case control study and a vector survey to determine the risk factors for acquisition of scrub typhus.

**Methods:**

A case control study with a 1:2 case control ratio was conducted over a 2 year period at a tertiary care centre and its surrounding districts in South India. Cases were children < 15 years with confirmed scrub typhus. Controls were age and locality matched community controls without fever. Demographic, environmental and behavioural risk factors were obtained in cases and controls by an interview and an environmental survey. A vector survey was also undertaken in the immediate vicinity of the cases.

**Results:**

Case Control study: 101 cases and 167 controls were analysed. On multivariate analysis, significant association was observed with environmental factors such as the presence of a water body within 100 m of the house (OR 3.56(1.36,9.75); p 0.011), cooking outside the house (OR 5.61 (1.51,23.01); p 0.011), owning pets (OR 3.33(1.16,9.09); p 0.031), and the presence of bushes within 5 m of the house (OR 2.78 (1.11,7.69); p 0.033). Of the behavioural factors, the child going to school by a vehicle (OR 3.12 (2.29,8.37); p 0.006) was associated with an increased risk. Drying clothes on a clothesline showed a trend towards protection from acquiring scrub typhus (OR 0.31 (0.08, 1.08); p 0.077).

Vector survey:26 rodents were trapped in as many houses. Trombiculid mites were isolated in 24 houses with 9(34.6%) being able to transmit scrub typhus. 254 trombiculid mites belonging to four species and two genera were collected. *Leptotrombidium deliense,* (33.5%). *Schoengastiella ligula,* (11.0%) of the total mite specimens collected. *S. ligula* always co-existed with *L. deliense.* The estimated Chigger index for *Leptotrombidium deliense* and *Schoengastiella ligula* was 3.27and 1.08 per animal respectively.

**Conclusions:**

Our study highlights risk factors for scrub typhus, some of which may be modifiable. A clean peri-domestic environment free of vegetation, drying clothes on a clothesline and cooking indoors may decrease the risk of scrub typhus.

## Author summary

Scrub Typhus is a re-emerging illness in the tropics with considerable morbidity and mortality. It is transmitted by mites to humans. A clear understanding of the risk factors for acquiring scrub typhus is essential to formulate preventive strategies. Our study was done to determine environmental and behavioural risk factors for acquiring scrub typhus in children. We compared children who had scrub typhus with age and locality matched children who did not have scrub typhus. We augmented our study with a vector survey by isolating mites from rodents around the homes of the children who had scrub typhus. In our analysis, we found certain environmental and behavioural factors that were associated with an increased risk and also found some factors which were protective. Our vector survey found that the mites capable of transmitting scrub typhus were found in and around the homes of the children who had scrub typhus. The details are presented and discussed in the study.

## Background

Scrub typhus is a mite borne zoonosis endemic in the so-called tsutsugamushi triangle with an estimated 1 billion people at risk and about 1 million cases occurring annually [1]. The disease is becoming increasingly wide spread with cases being recently reported from Africa, South America and the Middle East [2–4]. A distinct seasonality is noted with the disease occurring in the cooler months in South India [5]. Affected children usually present as a non-specific febrile illness with a typical eschar being present in about 40% of them [6]. Severe forms of the disease presenting as encephalitis with considerable mortality have also been described in children [7]. Delayed diagnosis due to the non-specific presentation, low index of suspicion and lack of availability of diagnostic tests results in significant morbidity and mortality [1, 8]. Antibiotic treatment has been shown to be effective in treating scrub typhus [9].

Scrub typhus is caused by *Orientia tsutsugamushi,* a gram negative intracellular pathogen. It is transmitted by the larval stage (chigger) of the trombiculid mite, usually of the genus *Leptotrombidium*. The mites are invisible to the naked eye and have a four-stage lifecycle: egg, larva, nymph and adult. The chigger needs a mammalian tissue meal to develop into the nymph. Only the bite of an infected chigger transmits disease. The other stages are free living in the soil [10, 11]. The bite leaves a characteristic black eschar that is useful for diagnosis, when found [12]. The mites act as the vector and the primary reservoirs for *O.tsutsugamushi* [13]. Once infected by feeding on the body fluid of mammals, they maintain the infection throughout their life stages by transovarial transmission and transtadial transmission [14]. Chigger mite populations can hence autonomously maintain their infectivity over long periods of time.

Scrub typhus is transmitted only by vectors and not from person to person. Therefore, an assessment of exposure to the infectious agent in order to determine the level of exposure in a setting and the risk of acquisition of disease over time is vital in understanding the disease. In addition, at the household level, determination of the modifiable environmental and behavioural factors that put a person at risk of contact with the vector and disease would aid the formulation of possible interventional strategies. Previous studies in adults have shown environmental and behavioural risk factors for acquiring scrub typhus with the highest risk in farm workers, those working in vegetable fields and hilly areas, and harvesting in autumn [15–17]. Children have limited mobility and different behavioural patterns and there are no similar studies in children. Hence, we performed a community based case control study to ascertain risk factors for acquisition of scrub typhus in children. We also conducted a vector survey on rodents captured from the vicinity of the houses of children with scrub typhus disease to determine the likely areas where children may have acquired their disease.

## Methods

### Case control study

The study period spanned 2 years from January 2015 to December 2016. Cases were children aged less than 15 years diagnosed to have scrub typhus at the Christian Medical College, Vellore (CMC) based on a positive InBiosScrub Typhus Detect™ IgM ELISA test (Inbios International Inc., Seattle, WA, USA) performed and interpreted as described previously [18]. Cases were included in the study if they resided within an 80 km radius of CMC and provided informed consent. Two age (+/− 1 year) and locality matched healthy controls were chosen for each case within a month of case recruitment. Age matching was done so that differences in behavioural patterns between ages are minimized. Location matching was done so that effect of differences in the macro-environment are negated. The house of the case was visited and closest street junction to the case’s house identified. A random number between 01 and 59 was then obtained to choose the control. The first digit denoted the direction of the street to be selected or a field hut (0-North, 1-East, 2-South, 3- West and 5- field hut). The second digit denoted the number of houses to be skipped in the chosen direction or the number of field huts to be skipped to choose a control. An age matched healthy control was then identified and chosen to take part in the study after obtaining informed consent. The same exercise was repeated to choose the second control. A recording form which included demographic, environmental and behavioural data was filled for both cases and controls. The recording form was filled by administering a questionnaire to a parent of the case or control and supplemented by a visual survey of the surroundings. Each control also had a blood sampling done for Scrub Typhus IgM and IgG to look for past or asymptomatic scrub typhus. All cases and controls houses were Geographic Information System (GIS) coded and plotted on a map to assess clustering. Socio-economic status scoring was done using the revised Kuppuswamy’s socio-economic status scale [19].

The potential risk factors assessed were determined by a pilot survey undertaken by two of the investigators. Sample size was calculated for a case to control ratio of 1:2 with the background exposure in controls expected to be 15%, to detect a factor with an odds ratio of 2 for cases with a 5% probability of type I error and 80% power. Ninety seven cases and 194 controls were needed.

Statistical analysis Analysis of data was performed using statistical software STATA version 13.1 (StataCorp, College Station, Texas, USA). As a first step, we excluded all controls who were either positive for scrub typhus IgM or IgG to ensure inclusion of only eligible controls. We used descriptive statistical methods (means, medians quartiles, standard deviations, frequencies and percentages)_to summarize all study variables. Secondly, we compared the distribution of environmental, behavioural and socio-demographic risk factors between cases and controls using chi-square or fisher’s exact tests, as appropriate. Finally, all factors with *P* ≤ 0.1 in the bi-variate analyses and other known prior risk factors were considered for inclusion in the multi-variable logistic regression analysis to identify independent risk factors for scrub typhus. Results from multivariable logistic regression analysis were presented as odds ratios with 95% confidence intervals (CI).

### Vector survey

The houses of all the cases in the case control study were visited by a field worker. Rodents and shrews were captured using Sherman traps of the size 3″ × 3″ × 10 ″ (W x H x L), designed for live capture of rats. The traps were baited with fried coconut. The traps were placed one hour before sunset and collected the next day morning. In each of the cases’ houses, 2 or 3 traps were placed inside and just around the houses. Once the rodent was trapped, the rodent was placed in a glass desiccator and a glass lid placed over the desiccator. Only one rodent was placed at a time in the desiccator. A single piece of unspun cotton was dipped in chloroform and then placed inside the glass desiccator. The animal is observed for cessation of voluntary movement and recumbency. A deep plane of anaesthesia was indicated by the lack of righting reflex when the container was tipped slightly and the respiratory rate was reduced to about 50% of the pre-anaesthetic rate (80–100 breaths/min). This usually happened in about 1 min in mice and 2 min in rats. If at any time the rodent had difficulty breathing (respiration becomes laboured, slows or stops), the desiccator was immediately opened and the rodent removed. Once the rodent was in deep plane of anaesthesia, it was removed from the desiccator and the animal’s mucous membrane colour, respiratory rate and withdrawal reflexes are checked. A noxious stimulus (toe pinch) was applied and if the animal responded to the toe pinch, it was returned to the desiccator for another 30 s. If the mucous membrane colour and respirations were normal and its withdrawal reflexes were absent, the rodent was ready for the mite collection. The ears and inter-digital spaces were swabbed with cotton tipped ear buds and the mite chiggers collected in an eppendorf tube with 70% alcohol. The hair was then combed on to a plastic tray and the mites collected in another eppendorf tube. If the rodents were trapped inside houses, they were released outside the house in the wild and if they were trapped outside, they were released back in the same place. Within a few minutes, the rodents were able to regain consciousness and run away. The eppendorf tubes were transported to the laboratory for identification of the mites and further testing. Mites were mounted in Hoyers medium, examined under the microscope and identified up to species level using standard taxonomical keys [20]. The chigger index (average number of chiggers per rodent) was estimated [21].

### Ethics statement

Ethics committee approval was obtained from the Institutional Review Board (Registration Number: ECR/326/INST/TN/2013 issued under Rule 122D of the Drugs & Cosmetics Rule 1945, Govt. of India) of Christian Medical College, Vellore to conduct the study. Institutional Animal Ethics Committee (IAEC) of the Christian Medical College, Vellore (Registration number: 88/PO/RcBi/SL/1999/CPCSEA registered with Committee for the Purpose of Control And Supervision of Experiments on Animals (CPCSEA), Ministry of Environment, Forests and Climate Change, Government of India) approval was also obtained for trapping of rodents to obtain mite chiggers. Written informed consent was obtained from a parent/guardian of all children who were enrolled in the study. Written assent was obtained from all children above the age of 8 years in addition to the parent/guardian consent. The rodents and shrews were captured using traps and anaesthetized to obtain mites from them. All rodents and shrews were released back in the same environment after obtaining the mites.

## Results

**Fig. 1:**
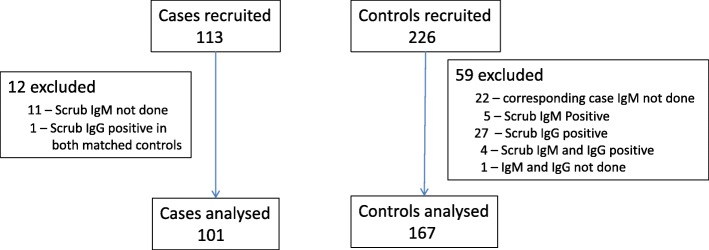
Flow chart of cases and controls recruited

There were 113 cases and 226 controls recruited in the study. Twelve cases and 59 controls were excluded from analysis. The main reasons for exclusion were scrub typhus IgM/IgG positivity in the controls, and loss of samples in the laboratory (11 cases and their corresponding 22 controls) as shown in Fig. 1. In all 101 cases and 167 controls were used in the final analysis.

All the cases belonged to three administrative districts viz., Chittoor district in Andhra Pradesh and Vellore and Tiruvannamalai districts in Tamil Nadu. The baseline demographic data of cases and controls are given in Table 1.

**Table 1:** Baseline demographic data of cases and controls

	Cases (%) N 101	Controls (%) N 167
Age < 5 years	50 (49.5)	81 (48.5)
Male	59 (58.4)	104 (62.3)
Father’s occupation - skilled	42 (41.6)	39 (23.4)
Highest family education atleast high school	74 (73.3)	107 (64.1)
Socio-economic status – middle or high	41 (40.6)	38 (22.8)

Many demographic, environmental and behavioural factors that were evaluated by matched pair analysis as shown in Table 2. Factors associated with a significant risk of scrub typhus on univariate analysis were the following: belonging to a middle or high socio-economic stratum, father having a skilled occupation, the presence of a water body within 100 m of the house in the form of irrigation canals or small lakes, living in a house with plastered walls, living in a house with more than 2 rooms, having furniture to sit and cots for sleeping, having piped drain for sullage, having a toilet, storing agricultural produce in the house, owning a pet, presence of bushes within 5 m of the house, using sitting on furniture, usually sleeping on a cot, and going to school by a vehicle. The only protective factor on univariate analysis was the child usually playing outside the house. On multivariate analysis including all factors with a *p* < 0.1, the only variables that were associated with significant risk were the presence of a water body within 100 m of the house (OR 3.56(1.36,9.75); p 0.011), cooking outside the house (OR 5.61 (1.51,23.01); p 0.011), owning pets (OR 3.33(1.16,9.09); p 0.031), the presence of bushes within 5 m of the house (OR 2.78 (1.11,7.69); p 0.033) and going to school by a vehicle (OR 3.12 (2.29,8.37); p 0.006). Drying clothes on a clothesline showed a trend towards protection from acquiring scrub typhus (OR 0.31 (0.08, 1.08); p 0.077).

**Table 2:** Univariate and multivariate analysis on demographic, environmental and behavioural factors associated with acquisition of scrub typhus

Variable	Univariate Analysis			Multivariate analysis		
	OR	95% CI	*p*-value	OR	95% CI	p
Demographic Factors						
Age group < 5 years	1.04	(0.64,1.71)	0.874			
Sex- Male	0.85	(0.51,1.41)	0.531			
Father occupation – Skilled	2.33	(1.37,4)	0.002	1.25	(0.21,3125)	0.663
Mother occupation – Skilled	0.67	(0.38,1.16)	0.157			
Highest family education –atleast high school	1.54	(0.89,2.7)	0.122			
Socio Economic Score – Middle or high	2.32	(1.35,4)	0.002	1.35	(0.45,3.57)	0.546
Environmental Factors						
Location of house – Adjoining field	1.56	(0.91, 2.7)	0.109			
Road leading to house – Paved	0.93	(0.55,1.56)	0.773			
Presence of water body within 100 m	2.65	(1.43,4.94)	0.002	3.56	(1.36,9.75)	0.011
Terraced house	1.37	(0.79,2.38)	0.254			
Cemented/tiled floor	2.08	(0.56,7.75)	0.275			
Plastered walls	2.76	(1.01,7.57)	0.048	8.03	(0.99,187.97)	0.091
Concrete roof	1.49	(0.86,2.6)	0.157			
House with > 2 rooms	2.33	(1.41,3.87)	0.001	1.18	(0.52,2.68)	0.678
Furniture used for sitting	1.83	(1.02,3.3)	0.044	1.53	(0.55,4.43)	0.413
Cot used for sleeping	2.18	(1.28,3.73)	0.004	1.52	(0.56,4.15)	0.401
Paved surroundings	1.43	(0.87,2.35)	0.156			
Piped drain for sullage	2.4	(1.32,4.35)	0.004	1.22	(0.43,3.54)	0.703
Cesspool around the house	1.26	(0.51,3.11)	0.611			
Cooking outside the house	1.92	(0.95,3.86)	0.068	5.61	(1.51,23.01)	0.011
Gas used for cooking	1.45	(0.78,2.7)	0.239			
Toilet present	1.75	(1.06,2.85)	0.027	1.19	(0.42,3.33)	0.731
Agricultural produce stored in house	2.22	(1.22,4)	0.008	1.2	(0.43,3.22)	0.742
Stored firewood present	0.97	(0.57,1.64)	0.911			
Wood piled in yard	1.00	(0.59,1.67)	0.99			
Owns live stock	1.2	(0.71,2)	0.482			
Owns pets	2.27	(1.19, 4.35)	0.013	3.33	(1.16,9.09)	0.031
Has contact with pets	0.67	(0.38,1.19)	0.169			
Rodents spotted within house	0.94	(0.55,1.64)	0.836			
Rodents spotted around the house	1.22	(0.41,3.7)	0.721			
Presence of bushes within 5 m of the house	2.27	(1.27,4.17)	0.006	2.78	(1.11,7.69)	0.033
Drying clothes on clothesline	0.49	(0.23,1.04)	0.064	0.31	(0.08, 1.08)	0.077
Child’s Behavioural Factors						
Usually partially dressed at home	0.71	(0.4,1.26)	0.241			
Wears footwear during play	1.73	(0.72,4.16)	0.218			
Plays outdoors usually	0.38	(0.21,0.68)	0.001	0.42	(0.14,1.23)	0.115
Usually sits on furniture	2.23	(1.02,4.86)	0.043	0.45	(0.13,1.48)	0.193
Usually sleeps on cot	2.23	(1.3,3.81)	0.004	1.53	(0.64,3.74)	0.341
Sleep outside house - Yes	0.99	(0.23,4.24)	0.991			
Uses toilet	1.51	(0.9,2.55)	0.118			
Usually bathes after play	1.92	(0.78,4.69)	0.153			
Usually changes clothes before sleep	1.39	(0.55,3.47)	0.484			
Goes to school by vehicle	4.38	(2.29,8.37)	< 0.001	3.12	(1.40,7.12)	0.006
Uses insect repellent	1.92	(0.81,4.54)	0.139			

**Fig. 2:**
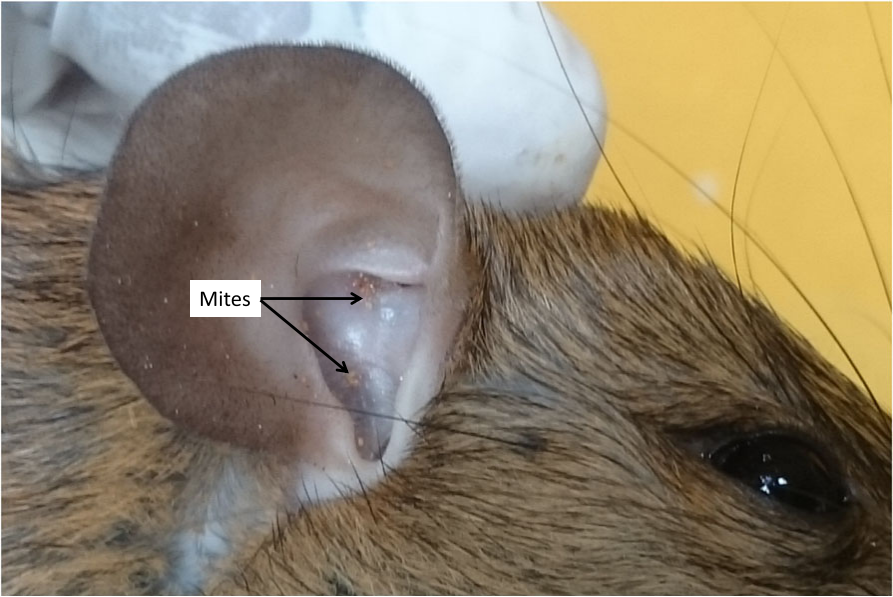
Mite colonies in the ear of a rodent

**Fig. 3:**
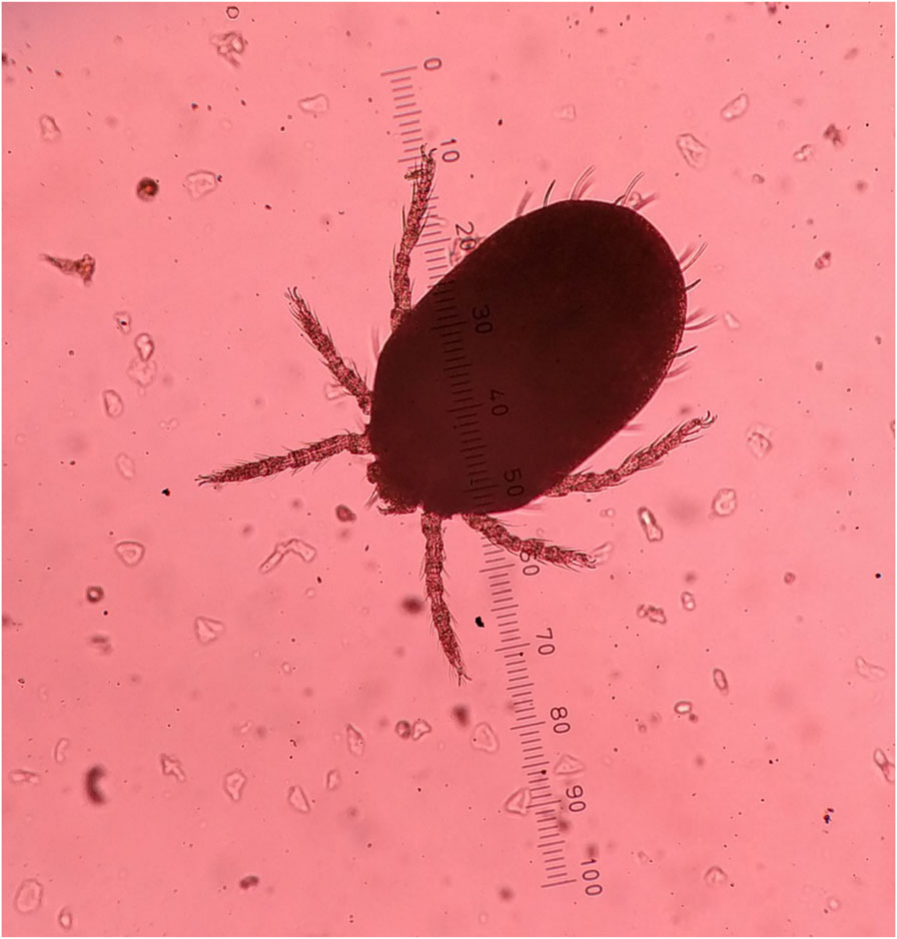
Chigger of a trombiculid mite under microscope on a 1 mm scale

Rodents were trapped in and around 26 of the cases’ houses. Trombiculid mites (Figs. 2 and 3) were isolated in 24 of them. Trombiculid mites capable of transmitting scrub typhus were isolated in 9 (34.6%) of the houses where the rodents were trapped. A total of 254 trombiculid mites belonging to four species and two genera was collected (Table 3). *Leptotrombidium deliense,* the major vector of scrub typhus pathogen [18] was the predominant species (33.5%) next to *Leptotrombidium insigne*. *Schoengastiella ligula,* the secondary vector of scrub typhus [21] constituted 11.0% of the total mite specimens collected and the species was isolated in 4 (15.4%). *S. ligula* always co-existed with *L. deliense.* The estimated Chigger index for *Leptotrombidium deliense* and *Schoengastiella ligula* was 3.27and 1.08 per animal respectively.

**Table 3:** Distribution of mites isolated

Mites isolated	Number of houses where mites isolated(%)	Number of mites isolated(%)
*Leptotrombidium deliense*	9 (34.6)	85 (33.5)
*Leptotrombidium insigne*	19 (73)	139 (54.7)
*Schoengastia sp.*	2 (7.7)	2 (0.8)
*Schoengastiella ligula*	4 (15.4)	28 (11)
No mites	2 (7.7)	
Total	26	254

## Discussion

Our study was conducted to determine demographic, environmental and behavioural risk factors for acquisition of scrub typhus in children. Similar studies have been done in adults, where the main risk factors were related to their occupation. Since occupation is not relevant and mobility is relatively restricted in children, our study brings out those risk factors which are related to the immediate environment where children live and their behaviour. Environmental factors seemed to be the most important in increasing the risk for scrub typhus compared to demographic and behavioural factors. The presence of a water body in the vicinity of the house, cooking outside the house, owning pets and the presence of scrub vegetation in the immediate surroundings of the house were all associated with an increased risk.

Of the environmental factors, the presence of a water body within 100 m of the house showed a 3.5 fold increase in the risk of scrub typhus. Small geographic regions which are high risk for humans to acquire scrub typhus include forest clearings, river banks and grassy regions. These regions which provide the optimal environment for the infected mites to thrive have been described as ‘scrub typhus islands’ [22]. It is likely that the presence of cases in the vicinity of a water body represents such an island. The presence of bushes in close vicinity of the houses is associated with a 2.8 fold risk of acquiring scrub typhus and is a modifiable risk factor. Trombiculid mites have been isolated from poorly maintained kitchen gardens [23] and chigger density has been associated with grass density [10]. Keeping the immediate surroundings of the houses clean and free of vegetation may decrease the risk of acquisition of scrub typhus especially in children.

Of the behavioural risk factors, cooking outside the house which is practised in certain rural south Indian households is associated with an over 5 fold risk of acquisition of scrub typhus. It is likely that the storage and use of material used for cooking becomes a favourable environment for rodents and rodent density is linked with increased transmission of scrub typhus [2, 24]. In our study, owning a pet confers a 2.3 fold risk of acquiring scrub typhus possibly by increasing the chance of a child coming into contact with a chigger. The chiggers that transmit scrub typhus use many small mammals to complete their life cycle [11]. Dogs have been found to be infected with *O. tsutsugamushi* and could well be a reservoir in the transmission cycle of scrub typhus [25]. Although household pets have not been demonstrated to harbour mites transmitting scrub typhus, our study has demonstrated significant association of pets within the patients compared to controls. Study of pets especially dogs and the likelihood of their harbouring vectors such as mites that have a potential for transmitting scrub typhus may need further study. Dogs were the predominant pets in our study too being present in 20.4% of the cases’ houses. Travelling to school by a vehicle was also associated with a 4.4 fold increase in risk of scrub typhus in our study which is counter intuitive. It is possible that children travelling to school by vehicle travel a greater distance from their homes with an increased risk of exposure outside of their homes in addition to their own peri-domestic environment. Use of a clothesline to dry clothes showed a trend towards being protective against acquiring scrub typhus (OR 0.31; p 0.077). It is a common practice in rural areas for washed clothes to be dried on scrub vegetation, which can increase the risk of a chigger being transported on to the child’s body when the clothing is being worn. Use of a clothesline to dry clothes may prevent such translocation of chiggers.

The vector survey done in our study by capturing rodents in the immediate vicinity of children who had scrub typhus disease has shown that 34.6% of houses where rodents were captured had the vector capable of transmitting scrub typhus. The estimated chigger (*L. deliense* and *S.ligula*) index was well above the critical level of 0.69 per rodent [21] in the villages surveyed indicating that the villages are receptive for scrub typhus transmission. Vector density is known to vary considerably depending on the season, vector species and rodent species [26, 27]. Many studies have trapped rodents in villages and found scrub typhus transmitting vectors, but have not been linked to cases of scrub typhus [10, 28, 29]. Our findings strengthen the argument that children could acquire scrub typhus close to their homes.

The main limitation of the study is the analysis with lesser number of controls than anticipated. Though the study recruited controls at a case control ratio of 1:2, 36 controls had to be excluded because of their seropositivity for scrub typhus. The seropositivity among the controls underlines that there is scrub typhus in these locations. The choice of parameters to compare between cases and controls were based on previous literature on similar studies and a field visit by the investigators. It is possible that some parameters may have been overlooked. The possible selection bias in the patients with higher SES seeking care at a tertiary centre and the absence of information on the health seeking behaviour among cases and controls is another limitation in the study. The vector survey identified the presence of chiggers capable of transmitting scrub typhus in the vicinity of the houses of children who had scrub typhus disease. Demonstration of *O. tsutsugamushi* in the chiggers would have strengthened the vector survey.

## Conclusions

In conclusion, our study highlights risk factors for scrub typhus, some of which may be modifiable. A clean peri-domestic environment free of vegetation, drying clothes on a clothesline and cooking indoors may decrease the risk of scrub typhus. Further studies are needed to determine the role of pets in the transmission of scrub typhus. With increase in the number and distribution of cases of scrub typhus in many countries, more studies are needed to evaluate the vector host interaction to institute public health measures for prevention.

## Data Availability

The data can be made available by the corresponding author. The email address of the corresponding author is winsleyrose@cmcvellore.ac.in

## Abbreviations

CI: Confidence Interval
GIS: Geographic Information system
SES: Socio-economic Status
